# Impact of diffusion-weighted imaging on agreement between radiologists and non-radiologist in musculoskeletal tumor imaging using magnetic resonance

**DOI:** 10.1016/j.ejro.2024.100590

**Published:** 2024-07-13

**Authors:** Gustav Lodeiro, Katarzyna Bokwa-Dąbrowska, Andreia Miron, Pawel Szaro

**Affiliations:** a Department of Radiology, Institute of Clinical Sciences, Sahlgrenska Academy, University of Gothenburg, Gothenburg, Sweden; b Department of Musculoskeletal Radiology, Sahlgrenska University Hospital, Gothenburg, Sweden

**Keywords:** Diffusion-weighted imaging, Magnetic resonance imaging, Sarcoma, Musculoskeletal tumor, Radiologist, Non-radiologist

## Abstract

**Background:**

Diffusion-weighted imaging (DWI) is widely used in neuroradiology or abdominal imaging but not yet implemented in the diagnosis of musculoskeletal tumors.

**Aim:**

This study aimed to evaluate how including diffusion imaging in the MRI protocol for patients with musculoskeletal tumors affects the agreement between radiologists and non-radiologist.

**Methods:**

Thirty-nine patients with musculoskeletal tumors (Ewing sarcoma, osteosarcoma, and benign tumors) consulted at our institution were included. Three raters with different experience levels evaluated examinations blinded to all clinical data. The final diagnosis was determined by consensus. MRI examinations were split into 1) conventional sequences and 2) conventional sequences combined with DWI. We evaluated the presence or absence of diffusion restriction, solid nature, necrosis, deep localization, and diameter >4 cm as known radiological markers of malignancy. Agreement between raters was evaluated using Gwet’s AC1 coefficients and interpreted according to Landis and Koch.

**Results:**

The lowest agreement was for diffusion restriction in both groups of raters. Agreement among all raters ranged from 0.51 to 0.945, indicating moderate to almost perfect agreement, and 0.772–0.965 among only radiologists indicating substantial to almost perfect agreement.

**Conclusion:**

The agreement in evaluating diffusion-weighted MRI sequences was lower than that for conventional MRI sequences, both among radiologists and non-radiologist and among radiologists alone. This indicates that assessing diffusion imaging is more challenging, and experience may impact the agreement.

## Highlights

• The agreement between raters in assessing musculoskeletal tumors on MRI was lower when diffusion imaging was included.
• Diffusion imaging's complexity may deter use; thus collaboration is essential for developing tumor imaging techniques.
• Clinicians should collaborate with experienced radiologists to improve musculoskeletal tumor evaluation accuracy.

## Graphical Abstract

**Figure:**
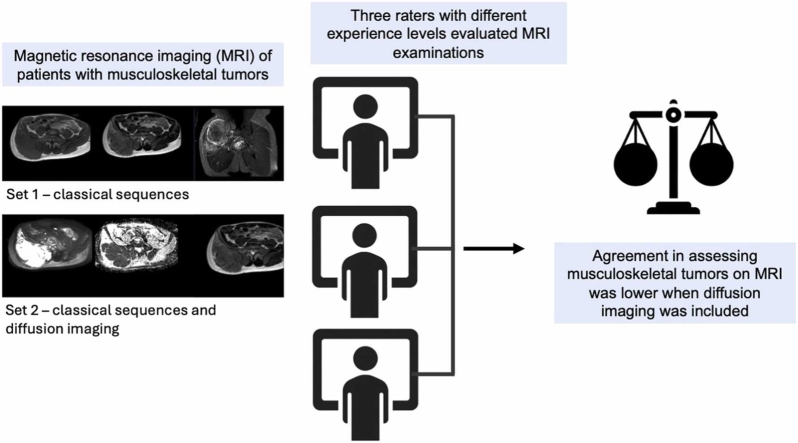

## 1 Introduction

The diagnosis of musculoskeletal tumors relies on the collaboration of clinicians, radiologists, and histopathologists [1]. Imaging diagnostics play a crucial role in determining the presence, location, size, and nature of the tumor [2]. Although radiologists primarily evaluate MRI, clinicians also review them. When clinicians better understand these images, they use them more frequently, enhancing clinical-radiological collaboration and improving clinical outcomes. Presenting patients to a multidisciplinary tumor board before any treatment increases adherence to clinical practice guidelines, improves surgical quality, and leads to better relapse-free survival rates with fewer complications and reoperations [3].

Differentiating between benign and malignant soft tissue tumors on MRI can be challenging due to overlapping features [4]. Malignant tumors often show increased cellularity, which may clearly restrict water diffusion, visible as diffusion restriction on imaging. Diffusion-weighted imaging (DWI) and apparent diffusion coefficient (ADC) values hold promise for distinguishing malignant from benign lesions without needing contrast injection, though its potential in larger patient cohorts remains underexplored [5], [6], [7]. However, not only malignant tumors show diffusion restriction. Since ADC is a quantitative sequence, measuring its values could, in the future, help differentiate between tumor types [8]. The use of a more quantitative assessment makes the evaluation of the study less subjective. In the future, this may help in applying diffusion in the design of artificial intelligence systems that aid in more objective differentiation of tumors.

The use of diffusion imaging may become an integral part of clinical protocols in the future. Currently, there is a scarcity of studies on the value of diffusion imaging in musculoskeletal tumors. However, employing standardized templates enables more systematic and structured reporting of findings, making it easier for recipients of radiological reports to compare and interpret the information [9], [10]. Similar research has been conducted for rectal cancer, where an increased focus on MRI staging has led to improved survival rates. This has created a need for better cooperation between radiologists and non-radiologists, highlighting the importance of systematic reporting [9], [10].

Several significant advantages of diffusion-weighted imaging include not requiring gadolinium-based contrast agents, which are commonly used in tumor examinations and may carry risks and potential side effects. Despite these benefits, DWI is seldom used in clinical applications for musculoskeletal tumors today, leaving several potential advantages unexplored [11].

To our knowledge, this is the first study aimed at evaluating the agreement between radiologists and non-radiologist in assessing MRI with diffusion for musculoskeletal tumors. This study aims to assess the impact of diffusion imaging on inter-rater agreement between radiologists and non-radiologist assessing musculoskeletal tumors.

## 2 Aim

This study aimed to evaluate the impact of including diffusion-weighted imaging (DWI) in MRI protocols on the agreement between radiologists and non-radiologist in assessing malign and benign musculoskeletal tumors.

## 3 Material and methods

### 3.1 Flow

We reviewed MRI examinations of patients consulted at our institution (which is the tertiary center for musculoskeletal tumors) over the past four years. Patients diagnosed with musculoskeletal tumors, whose MRI examinations included diffusion imaging, were included in the study consecutively. The first and last authors excluded patients whose examinations were with low-quality or artifacts, as well as those performed during treatment or post-operation. MRI sequences were separated into two sets. Set 1 included conventional sequences (T1-weighted with and without contrast, T2-weighted, Short Tau Inversion Recovery (STIR)) but *without* diffusion imaging. Set 2 included conventional sequences (T1-weighted without contrast, T2-weighted, STIR) *with* diffusion imaging but *without* contrast. Raters evaluated Set 1 and 2, and consensus between raters was the final result.

### 3.2 Raters

All raters participated in a basic training session led by the last author, focusing on evaluating diffusion-weighted imaging and the study protocol to ensure consistent and accurate assessments. The training session lasted 3 hours and included 5 example patients: 3 with Ewing sarcoma, 1 with osteosarcoma, and 1 with schwannoma, all outside the study cohort. After the training session, raters had two weeks to complete their rating protocols. No help or assistance was available during this period. Raters assessed the examinations alone. The assessment period was between February 2nd and February 16th, 2024.

Three raters participated in the study: non-radiologist - a medical student in their 10th term (Rater A, first author), a musculoskeletal radiologist with six years of experience (Rater B, second author), and a musculoskeletal radiologist with ten years of experience and certification by the Swedish Medical Association (Rater C, last author). Rater C had the most experience with DWI, followed by Rater B, with Rater A having almost no previous experience. All participants received the research protocols.

*Rationale for Inclusion of Non-Radiologist*: Including a medical student as a non-radiologist reflects real-world clinical practice, where multidisciplinary teams often include medical doctors who do not have experience in interpreting imaging.

### 3.3 MRI examinations

The examinations included in the study come from different scanners (General Electric, Philips, and Siemens) with magnetic fields of 1.5 T and 3.0 T. The MRI examinations were divided into two Set 1 and Set 2 – described in the section Flow. Raters evaluated Sets 1 and 2 at a dedicated radiological station within a four-week interval, in a randomized order. Examination sets with the same order of sequences were saved under two separate lists and displayed for raters in random order. Thus, all raters had the same collection of sequences. The raters could scroll through the sequences as needed. We included the evaluation of diffusion using b-values: b=0, b=100, and b=800. Raters were blinded to previous assessments, diagnoses, and clinical data.

### 3.4 Evaluation criteria

In Set 1 we included the following variables: 1) Solid tumor: defined as contrast-enhancing abnormal mass. 2) Necrosis is defined as non-contrast-enhancing foci in the tumor. 3) Deep-seated defined as a tumor located beneath the fascia. 4) Is the size greater than 4 cm at least on one plane when assessed on T1-weighted images with contrast. For Set 2 we evaluated the presence of the diffusion restriction was defined as hyperintensity on diffusion-weighted images (DWI) with a corresponding area of hypointensity on apparent diffusion coefficient (ADC) images. The diffusion restriction was assessed only visually as either present or absent.

### 3.5 Statistical analysis

Agreement between raters in the study was calculated using Gwet’s AC1 coefficients [12] on AgreeStat® 360 and interpreted using Landis and Koch [12], [13]. Differences in the presence of malignancy signs between malignant and benign lesions were evaluated using the chi-square test. A p-value < 0.05 was considered significant. Sample sizes were partly restricted by the available patient population. Estimating the sample size for this exploratory study was challenging due to the limited literature and the retrospective nature of many previous studies. Based on the literature, we aimed to include a minimum of 36 patients [14].

### 3.6 Ethic review

The study was approved by the Swedish Ethical Review Authority number 2023–07231–01.

## 4 Results

**Fig. 1:**
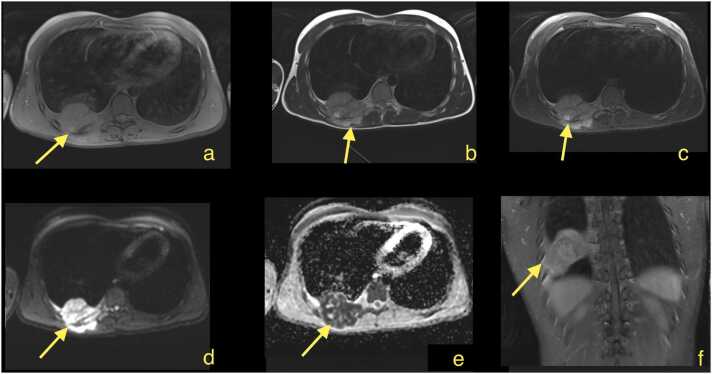
23-year-old patient with Ewing sarcoma. Magnetic resonance imaging showed a solid tumor in the thoracic wall measuring more than 4 cm, with discrete central necrosis.: A - T1-weighted with fat suppression, b - T2-weighted, c - T1-weighted with gadolinium contrast, d - diffusion-weighted imaging (DWI, b 800), e - apparent diffusion coefficient (ADC), f - T1-weighted with gadolinium contrast and with fat suppression.

We reviewed 1002 MRI examinations and identified 14 patients with Ewing sarcoma (Fig. 1), 14 patients with osteosarcoma, and 14 patients with benign tumors who underwent MRI with diffusion imaging (Table 1). Three patients were excluded from the study due to artifacts and missing T2-weighted sequences necessary to assess the included variables. Ultimately, we included 14 patients with Ewing sarcoma, 11 patients with osteosarcoma, and 14 patients with benign tumors (4 schwannomas, 2 lipomas,1 myxoma, 1 lipoblastoma and 6 benign conditions including reactive changes and inflammations), totaling 39 patients in the study. Patients were included consecutively. The mean age was 38.5 years (min 8, max 89, standard deviation 23.5 years). The cohort included 23 females and 16 males.

**Table 1:** Presence of malignancy signs in analyzed tumors.

		**Solid tumor**	**Necrosis**	**Deep localization**	**One diameter is more than 4 cm**	**Diffusion restriction**
**All tumors**		33	23	32	29	24
		89.2 %	62.2 %	86.5 %	78.4 %	72.7 %
**Malign tumors**	**All malign tumors**	21	18	21	20	20
		84.0 %	72.0 %	84.0 %	80.0 %	80.0 %
	**Ewing sarcoma**	12	10	12	11	11
		85.7 %	71.4 %	85.7 %	78.6 %	78.6 %
	**Osteosarcoma**	9	8	9	9	9
		81.8 %	72.7 %	81.8 %	81.8 %	81.8 %
**Benign tumors**		12	5	11	9	4
		85.7 %	35.7 %	78.6 %	64.3 %	28.6 %
**Malign vs. benign**		<.001	<.001	<.05	<.001	<.001

Tumors included in our study were located in the extremities, including the pelvis (n=29), thoracic wall (n=6), and abdominal wall (n=4).

The most common features of malignant tumors on conventional sequences were a solid nature and deep localization (91.3 %, Table 1). Nearly all malignant tumors were larger than 4 cm (87 %) and exhibited diffusion restriction (80 %). Most tumors also displayed necrosis (72 %, Table 1, Fig. 1). Diffusion restriction was visible in almost all analyzed cases of Ewing sarcoma and nearly all cases of osteosarcoma (90 %, Table 1).

Diffusion restriction was observed in approximately 75 % of the tumors, as shown in Table 1.

Most benign tumors were solid (85.7 %) and located deep (78.6 %). Diffusion restriction was rarely observed (28.6 %), as shown in Table 1.

Characteristics such as a solid nature, presence of necrosis, deep location, size larger than 4 cm in one dimension, and diffusion restriction were more commonly seen in malignant tumors, which was statistically significant, as indicated in Table 1.

### 4.1 Inter-rater agreement

The agreement among all raters ranged from 0.51 to 0.945, which Landis and Koch [13] classify as moderate to almost perfect. The highest agreement in Set 1 evaluations was observed for size assessment, while the lowest was for necrosis assessment (Table 2). In Set 2 evaluations, the agreement for diffusion restriction was 0.51 which is moderate according to Landis and Koch [13] (Table 2).

**Table 2:** Intra and inter-agreement of all tumors, Agreement in the study was assessed using Gwet’s AC1 coefficients.

	**All raters: non-radiologist and radiologists**				
**Set**	**Feature**	**Coefficient**	**Standard error**	**95 % CI**	**P-value**
**Set 1**	**Solid tumor**	0.815	0.067	(0.679,0.951)	<.001
	**Necrosis**	0.704	0.091	(0.519,0.889)	<.001
	**Deep-seated tumor**	0.927	0.043	(0.841,1)	<.001
	**Size more than 4 cm**	0.945	0.040	(0.864,1)	<.001
**Set 2**	**Presence of diffusion restriction**	0.510	0.105	(0.297,0.723)	<.001
	**Subgroup: only radiologists**				
	**Feature**	**Coefficient**	**Standard error**	**95 % CI**	**P-value**
**Set 1**	**Solid tumor**	0.898	0.061	(0.775,1)	<.001
	**Necrosis**	0.850	0.085	(0.678,1)	<.001
	**Deep-seated tumor**	0.965	0.036	(0.891,1)	<.001
	**Size than 4 cm**	0.959	0.041	(0.876,1)	<.001
**Set 2**	**Presence of diffusion restriction**	0.772	0.102	(0.565,0.98)	<.001

In the subgroup analysis of radiologist raters, the agreement was higher compared to all raters. According to Landis and Koch [13], the agreement for diffusion restriction was 0.772 which is substantial, and for other features, it was almost perfect (Table 2).

## 5 Discussion

Our study found that when diffusion imaging was part of the MRI examination, the agreement between raters decreased. This decline was observed not only among all raters but also among radiologists. To our knowledge, this is the first study evaluating the agreement between radiologists and non-radiologist in assessing MRI examination with diffusion imaging for musculoskeletal tumors. This is an important area of research because diffusion imaging holds significant potential for diagnosing musculoskeletal tumors due to its ability to provide functional information. However, its lower anatomical resolution and other sorts of imaging compared to classical sequences can make it challenging for clinicians to understand and evaluate effectively.

Our findings highlight the need for enhanced collaboration between radiologists and clinicians to improve the assessment of diffusion imaging. Such collaboration is essential for the proper development and integration of advanced imaging methods in clinical practice. By working together, radiologists and clinicians can ensure that diffusion imaging is utilized to its fullest potential, ultimately improving the diagnosis and treatment of musculoskeletal tumors.

We utilized Gwet’s AC1 to evaluate the agreement between raters because the AC1 statistic overcomes the limitations of kappa, which is sensitive to trait prevalence and rater classification probabilities—a phenomenon known as the kappa paradox [15]. AC1 offers a more robust measure of interrater reliability [16]. Gwet’s AC1 has demonstrated greater stability in inter-rater reliability coefficients compared to Cohen’s Kappa. Moreover, Gwet’s AC1 is less influenced by prevalence and marginal probabilities than Cohen’s Kappa [15].

Diffusion images have low resolution, are blurry, pixelated, and can be difficult to assess for both inexperienced radiologists and clinicians. Unfortunately, due to the nature of these images, there is a risk that despite the advantages of this imaging technique, it may not be widely adopted for musculoskeletal tumor imaging. Diffusion imaging shows random microscopic movement of primarily water molecules within the body, occurring both inside and outside of cells [17], making it a functional sequence [18], [19] rather than anatomical, which makes it somewhat challenging to differentiate anatomical structures solely on diffusion images. The apparent diffusion coefficient (ADC) provides a quantitative measure of diffusion restriction, depicted as an ADC map where each voxel represents a calculated diffusion rate in mm²/s. The ADC map, derived from DWI, quantifies diffusion restriction, aiding in the evaluation of tissue as malignant or benign. Additionally, the ADC map visually indicates diffusion restriction: dark areas signify diffusion restriction, while bright areas denote the absence of it [20]. However, in clinical practice, diffusion images are not evaluated separately but in conjunction with anatomical images. Therefore, in our study, raters evaluated the entire MRI examination along with DWI and anatomical images.

In our study, the agreement between raters in assessing diffusion restriction was lower when the group included non-radiologist. In other studies using diffusion but regarding other systems that musculoskeletal, the agreement between raters was slightly higher, ranging from moderate [21], to substantial, or almost perfect [22]. This indicates that the evaluating of diffusion imaging is not easy and requires clinical experience. Our observation may probably be due to the selection of radiologists who have experience in musculoskeletal radiology compared to non-radiologist. It can be concluded that professional experience in diffusion imaging assessment is very important, however, there is a lack of studies in the field of musculoskeletal radiology regarding the role of experience in assessing DWI examinations. Therefore, it seems appropriate to introduce a systematic assessment of MRI examinations and, where possible, objective, and easy classification systems [23]. In other parts of radiology, studies have been conducted where it has been proven that updating classifications and simplifying them results in increased agreement between raters regardless of experience [24]. In the field of musculoskeletal radiology, a study was conducted on the assessment of dynamic MR examination of the lumbar spine which demonstrates that the experience of the evaluator has a low impact on the assessment of spinal instability if correct classification is used [25]. The researchers had almost perfect agreement among themselves, indicating that the examinations were assessed similarly. We evaluated four basic features of malignancy and the presence of diffusion restriction. A clear examination assessment system and a simple protocol can improve agreement between raters, confirming previously conducted observations in the assessment of ultrasonographic and MRI examinations in other parts of the body [26].

Based on our findings, it is important to note that the assessments performed by non-radiologist in our study were lower than those by radiologists. A potential reason for this discrepancy is the difference in experience levels between our non-radiologist and those in the other study, which evaluated inter- and intraobserver variability in lymph node size, apparent diffusion coefficient (ADC) measurements, and morphological characterization among both inexperienced and experienced radiologists [14]. All raters were radiologists in training (residents) and specialist radiologists. The higher prevalence of rectal cancer means that even less experienced residents have encountered it, at least on CT scans, whereas the tumors included in our study are very rare and unusual. This rarity could contribute to the differing results. Additionally, our protocol included a visual assessment of diffusion, while the Grimm et al. [14] study focused on numerical measurements. This methodological difference could impact inter-rater agreement, making it less dependent on the rater's experience. This distinction highlights the need for further studies incorporating the measurement of diffusion values to potentially enhance inter-rater reliability.

Diagnostic of musculoskeletal tumors is multidisciplinary, and radiology is usually involved. Imaging begins with an X-ray examination, which in the case of skeletal changes may suggest a diagnosis. In many cases, further diagnostics with magnetic resonance imaging are necessary [27]. The radiologist evaluates the examinations, but the clinical partners are involved also in imaging assessment. Our experience indicates that clinicians prefer to evaluate images they understand, often those with high-resolution and easy-to-assess features, such as T2-weighted images. However, with sufficient experience, evaluating other sequences like diffusion imaging can become a very valuable diagnostic method in tumor diagnostic. Ewing sarcoma and osteosarcoma are an inseparable pair in differential diagnostic on imaging. The Ewing sarcoma family of tumors is a group of small round blue cell tumors that are histogenetically similar, often seen as a big soft tissue tumor with some bone involvement, however, there are types that are totally extraskeletal. Ewing sarcoma is the second most common malignancy in childhood [28]. Osteosarcoma is a malignant bone-forming tumor that is the second most common primary bone tumor after multiple myeloma in the skeletal system. Its occurrence is about 20 % of all primary bone tumors [29]. In children, osteosarcoma is considered the most common primary bone tumor [29]. However, distinguishing Ewing sarcoma from osteomyelitis can be also very difficult. Especially osteomyelitis, which is low virulent, can be a major diagnostic challenge for both radiologists and clinicians [30].

Our study revealed that diffusion-weighted imaging is rarely used in musculoskeletal diagnostic and only a quarter of the Ewing sarcoma cases had performed a DWI sequence and in half of the cases of osteosarcoma, this confirms that DWI is not commonly used when examining musculoskeletal tumors. This could have several different explanations. Firstly, the official recommendations by the Scandinavian Sarcoma Group do not include diffusion-weighted imaging in MRI examination [27]. This could partly be due to that the recommendations are from 2012 and are not updated with the development in MRI technology. Secondly, the lack of research within the field could contribute to the lack of knowledge and create uncertainty about how to interpret DWI. At the time of the study, PubMed shows a total of 66 results when searching for published articles with MRI+DWI+Sarcoma [31], compared to 2083 results when searching for MRI+DWI+Brain [32] (which is the current main use for DWI). Furthermore, the studies of Ewing sarcoma with DWI are almost nonexistent, with only 6 articles published on PubMed [33], while osteosarcoma has nearly double that [31]. Several of the studies mentioned have been conducted with a different methodology and are not comparable with the aims and purpose of this study. The combination of a lack of research, experience, and old recommendation protocols could make it difficult for radiologists and physicians to motivate performing additional sequences. With the current recommendations and research, the only motive for actually performing a DWI today when handling patients with musculoskeletal tumors is at the discretion of the responsible physician or radiologist.

Our study has several limitations. Conducted in a single center, it aimed to mirror the everyday clinical environment for radiologists. We included raters with varying experience levels to reflect the real-world applicability of DWI assessment. The rarity of Ewing sarcoma made achieving an adequate sample size challenging; future studies should involve multiple sarcoma centers for larger samples. The retrospective design may have introduced biases in data collection and analysis. Variations in b-values across different MRI machines were noted, though all included b0 and b800 as well as ADC, and had a magnetic field strength of at least 1.5 T. With only a few raters, inter-agreement analysis was limited; future studies need more raters to establish accurate agreement statistics. There is no consensus on when to use diffusion-weighted imaging, leaving it to the individual radiologist. The similarities between osteosarcoma and Ewing sarcoma also challenge both DWI and conventional imaging. Diffusion restriction was noted based on visual assessment, not quantitative measures. None of the raters were orthopedic surgeons. Despite these limitations, the clinical environment ensured real-world relevance. Further prospective research is needed to fully explore the potential of diffusion MRI in musculoskeletal diagnostics.

## 6 Conclusion

The agreement between raters in assessing musculoskeletal tumors on MRI was lower when diffusion imaging was included in the MRI protocol compared to MRI without diffusion imaging. The nature of diffusion imaging may discourage clinicians from readily using it. However, given its clinical value and the results of our study, we believe that better collaboration is crucial for the development of tumor imaging. Clinicians and radiologists inexperienced in assessing diffusion should work closely with radiologists who are experienced in diffusion imaging. This radiological-clinical collaboration can enhance the quality of diagnostics and improve clinical outcomes for patients with musculoskeletal tumors.

## Ethics approval and consent to participate

The Swedish Ethical Review Authority approved the study and waived the need for informed consent due to its retrospective nature (number 2023–07231–01). The study was conducted in compliance with the Declaration of Helsinki. The anonymization of patient data ensured data protection following the European General Data Protection Regulation. The study was performed in accordance with relevant guidelines and regulations.

## Funding

This project received no funding.

## CRediT authorship contribution statement

**Gustav Lodeiro:** Writing – review & editing, Writing – original draft, Methodology, Investigation, Formal analysis, Data curation, Conceptualization. **Katarzyna Bokwa-Dąbrowska:** Writing – review & editing, Writing – original draft, Visualization, Methodology, Formal analysis, Data curation. **Andreia Miron:** Writing – original draft, Visualization, Data curation. **Pawel Szaro:** Writing – review & editing, Writing – original draft, Validation, Supervision, Methodology, Formal analysis, Data curation, Conceptualization.

## Declaration of Competing Interest

The authors declare that they have no known competing financial interests or personal relationships that could have appeared to influence the work reported in this paper.
